# Supplementary material for: Bayesian learning of chemisorption for bridging the complexity of electronic descriptors
Source: Nat Commun. 2020 Nov 30;11:6132. doi: 10.1038/s41467-020-19524-z (PMC7705683; doi:10.1038/s41467-020-19524-z)
Supplement: Supplementary file 1 — Supplementary Information [file 41467_2020_19524_MOESM1_ESM.pdf]

Supplementary Materials: Bayesian Learning of Chemisorption for Bridging the Complexity of Electronic Descriptors

Siwen Wang, Hemanth Somarajan Pillai, Hongliang Xin

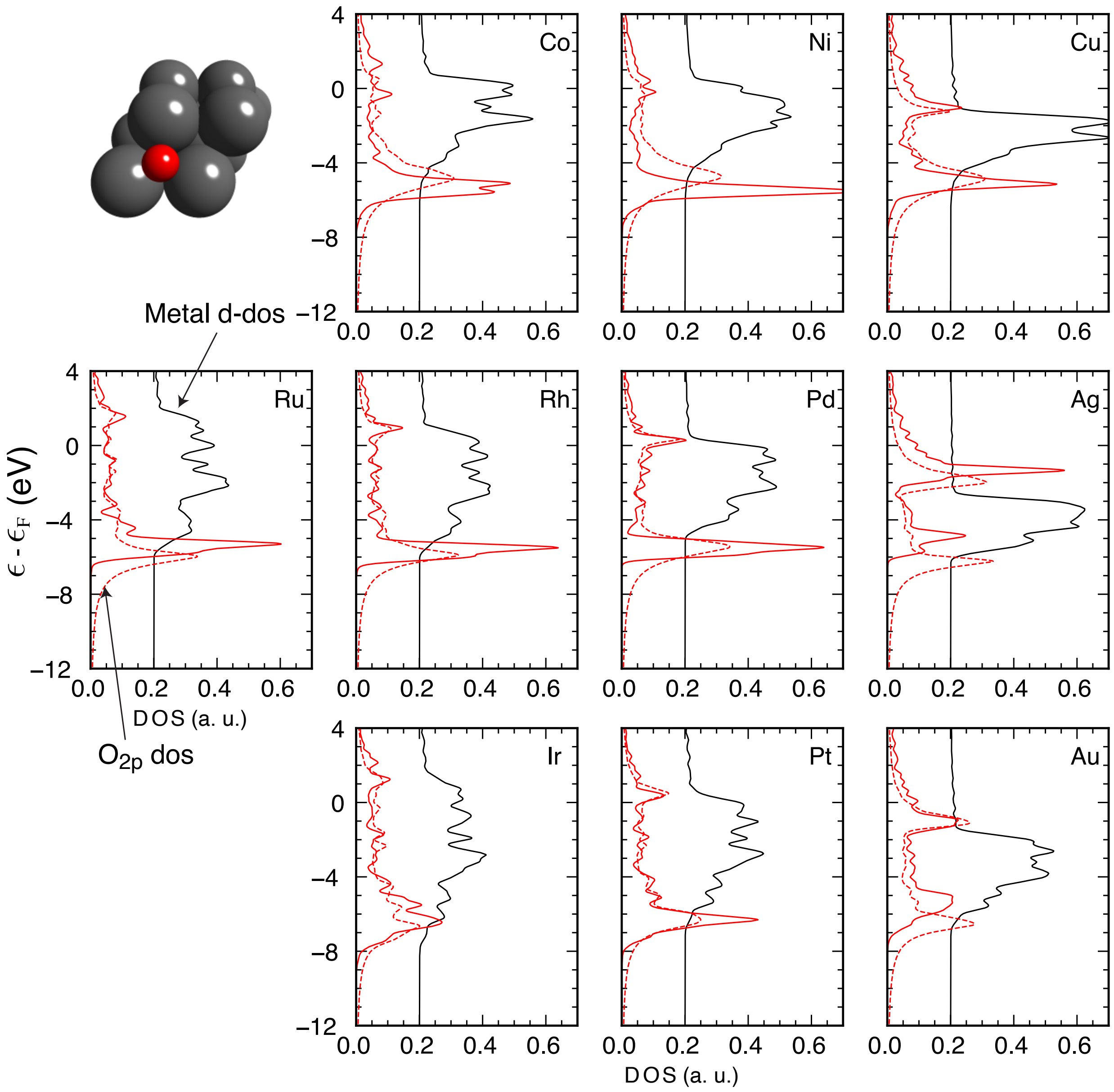

Supplementary Figure 1. Projected O_{2p} density of states from DFT calculations (solid) and model prediction (dashed) using the Bayesian-learned posterior means of single-orbital model parameters. Metal *d*-states are also shown.

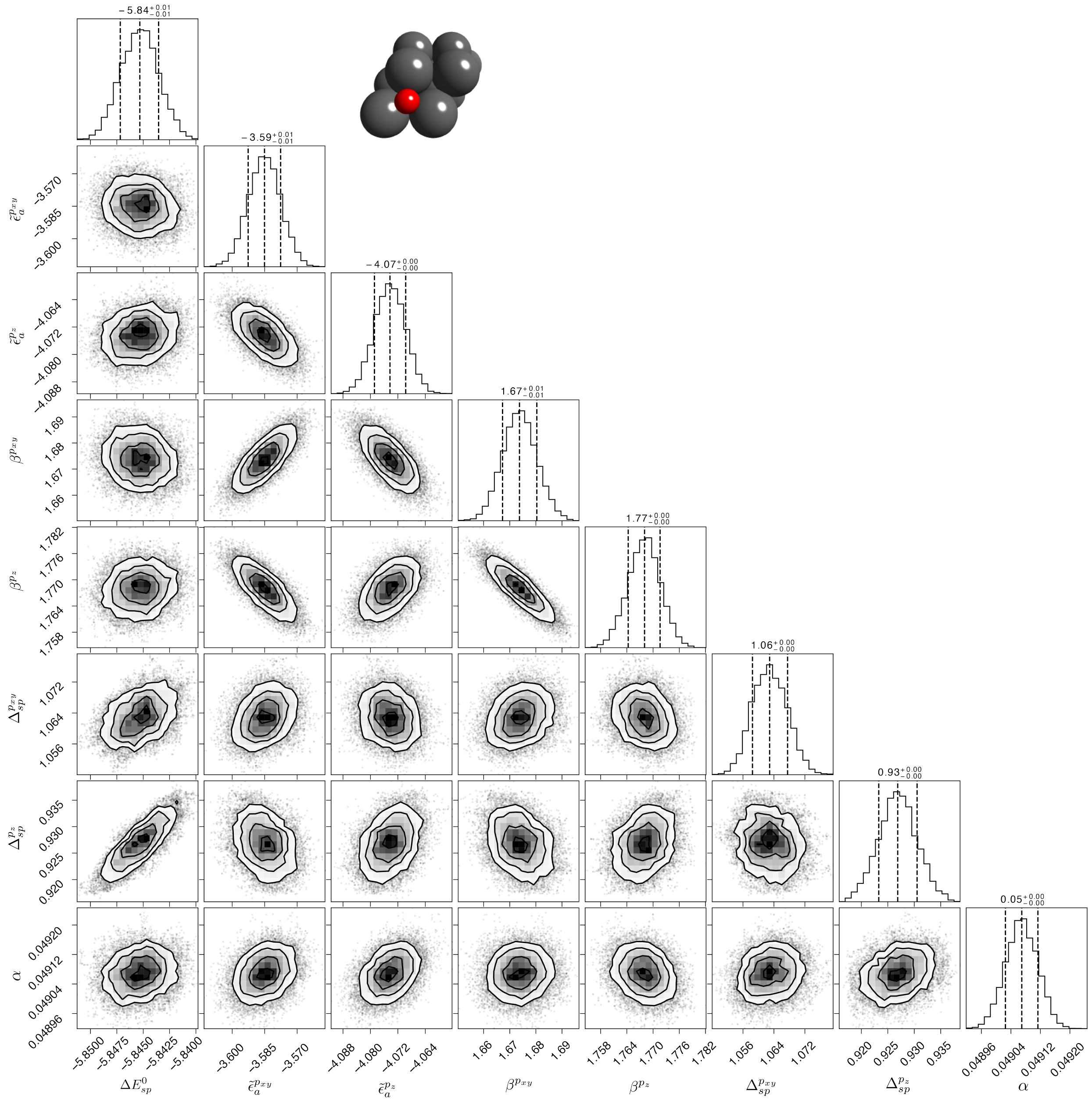

Supplementary Figure 2. The co-variance of the joint posterior distribution for each parameter pair and 1D histogram of multi-orbital model parameters (ΔE_0, $\varepsilon_a^{p_{xy}}$, $\varepsilon_a^{p_z}$, $\beta^{p_{xy}}$, β^{p_z}, $\Delta_0^{p_{xy}}$, $\Delta_0^{p_z}$, and α) from MCMC simulations for *O adsorption at the fcc-hollow site of the close-packed, pristine transition-metal surfaces.

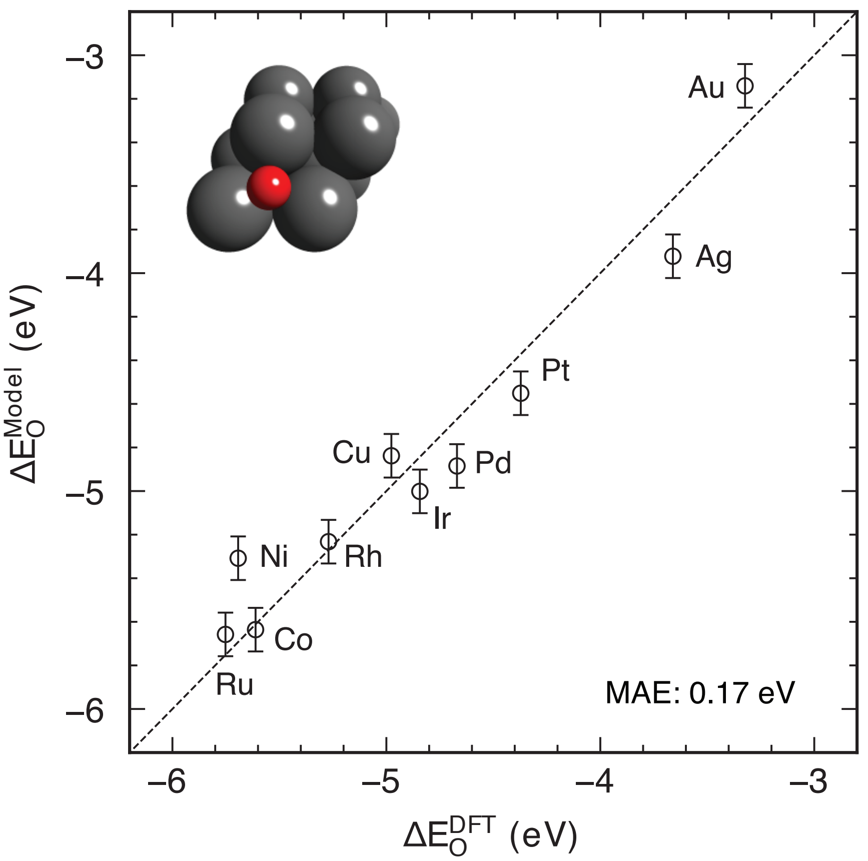

Supplementary Figure 3. DFT-calculated *O adsorption energies at metal surfaces vs. model prediction using the posterior distribution of multi-orbital model parameters ($\vec{\theta}$, $\vec{\sigma}$). Error bars represent the standard deviation of prediction based on Bayesian sampling.

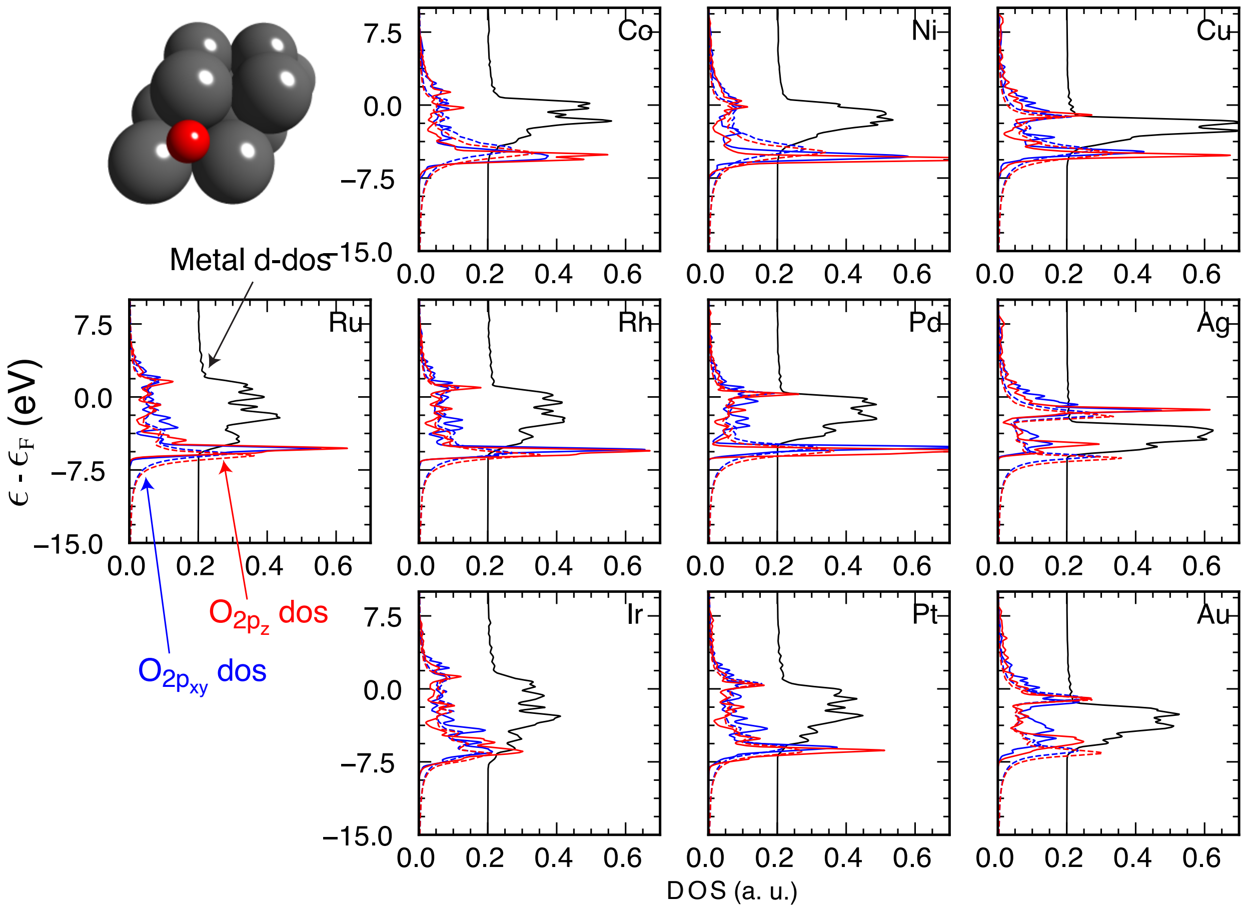

Supplementary Figure 4. Symmetry resolved projected O_{2p} density of states from DFT calculations (solid) and model prediction (dashed) using the Bayesian-learned posterior means of multi-orbital model parameters. Metal d-states are also shown.

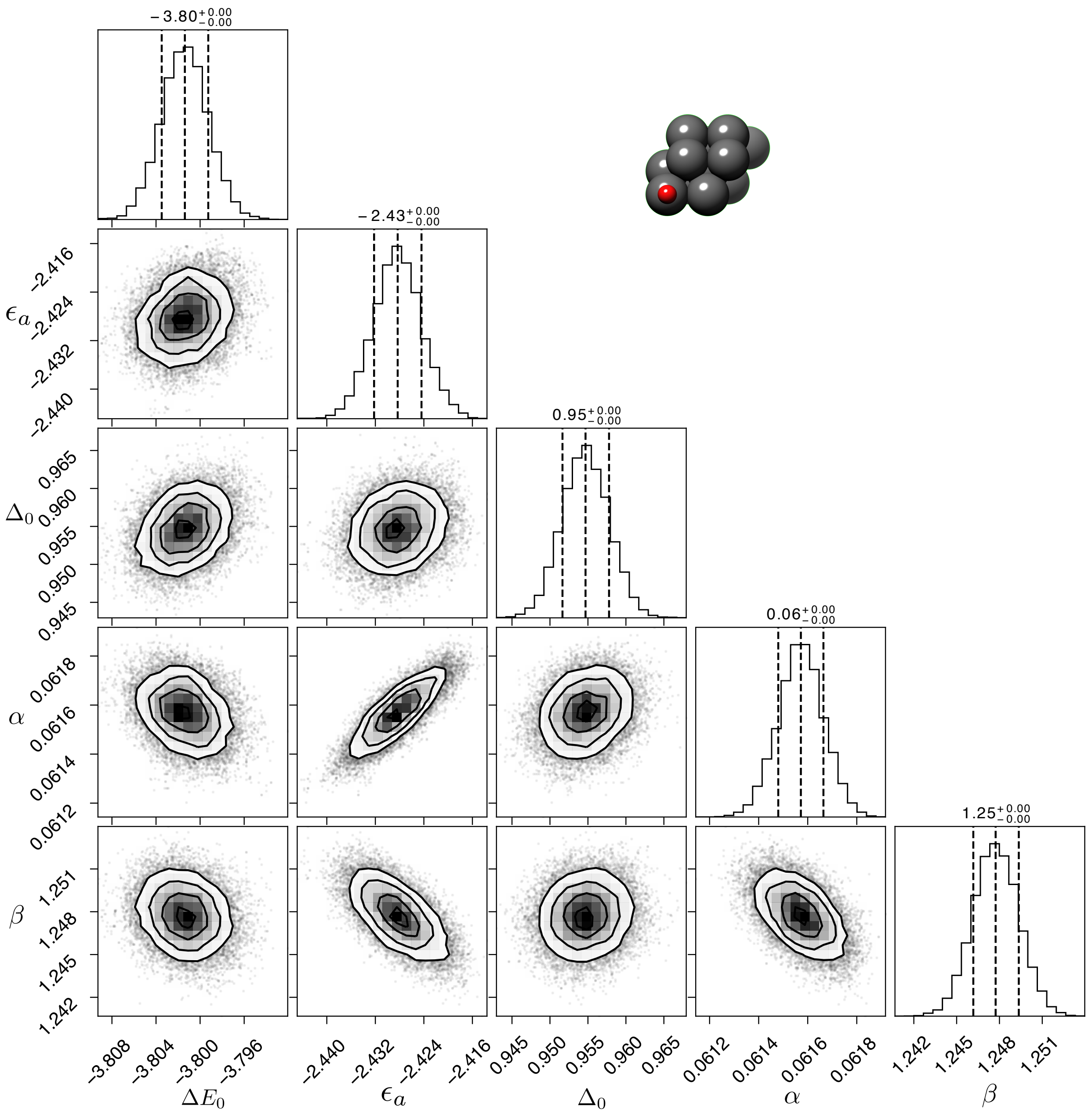

Supplementary Figure 5. The co-variance of the joint posterior distribution for each parameter pair and 1D histogram of single-orbital model parameters for oxygen adsorbed on atop (ΔE_0, ε_a, Δ_0, α, and β) from MCMC simulations for *O adsorption at the atop site of the close-packed, pristine transition-metal surfaces.

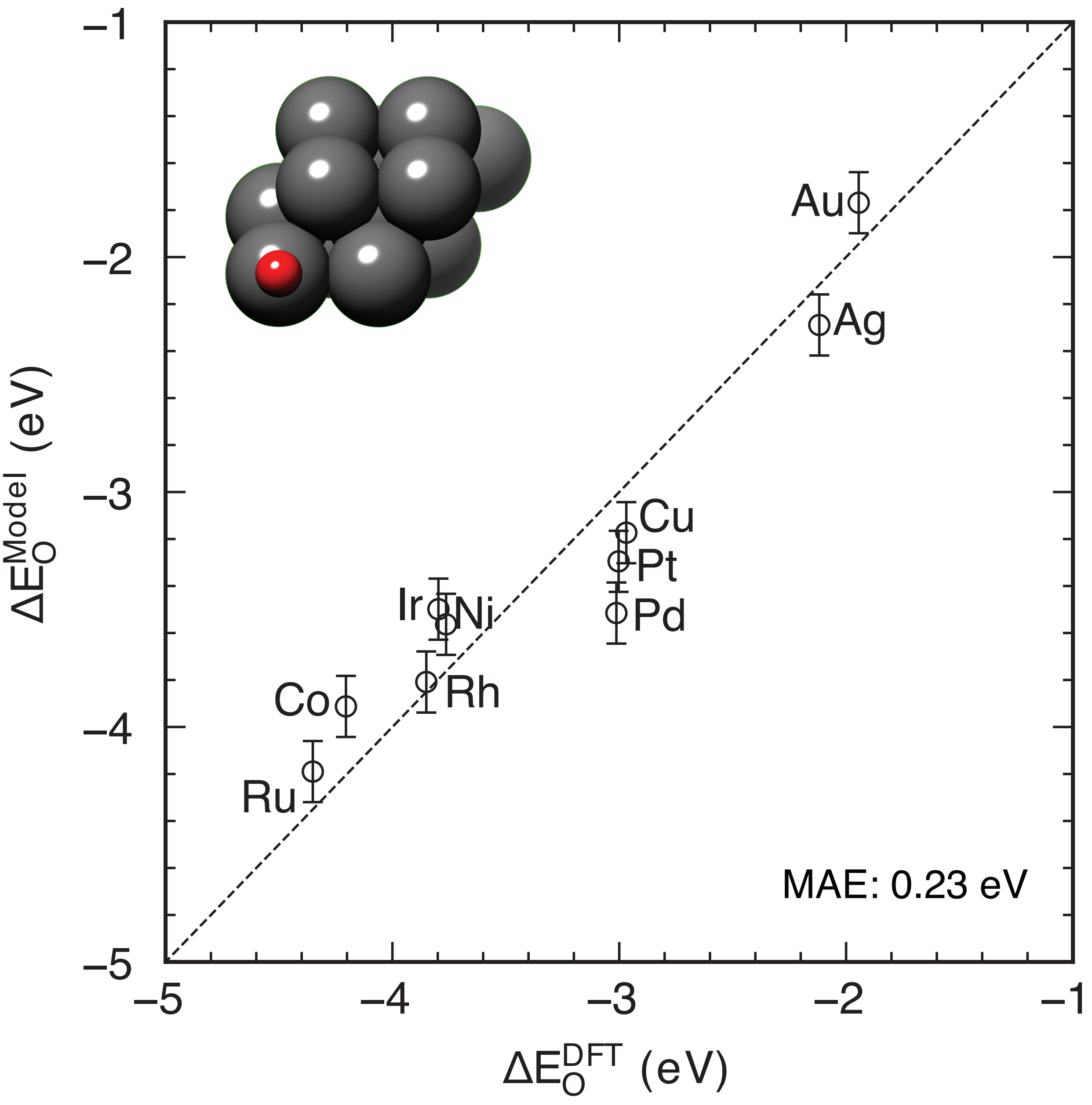

Supplementary Figure 6. DFT-calculated *O adsorption energies at the atop site of metal surfaces vs. model prediction using the posterior distribution of single-orbital model parameters $(\vec{\theta},\ \vec{\sigma})$. Error bars represent the standard deviation of prediction based on Bayesian sampling.

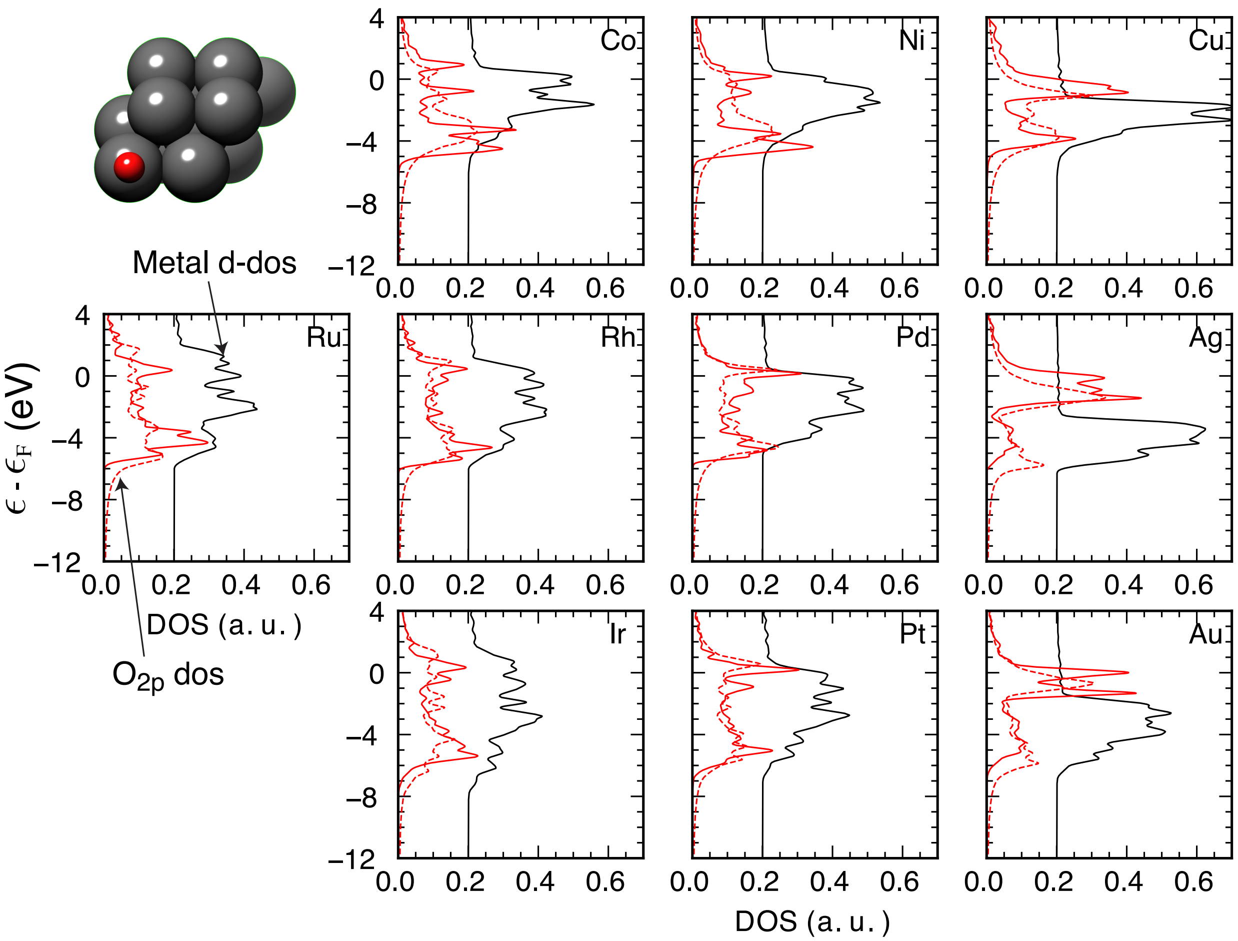

Supplementary Figure 7. Symmetry resolved projected O_{2p} density of states from DFT calculations (solid) and model prediction (dashed) for the atop site using the Bayesian-learned posterior means of single-orbital model parameters. Metal *d*-states are also shown.

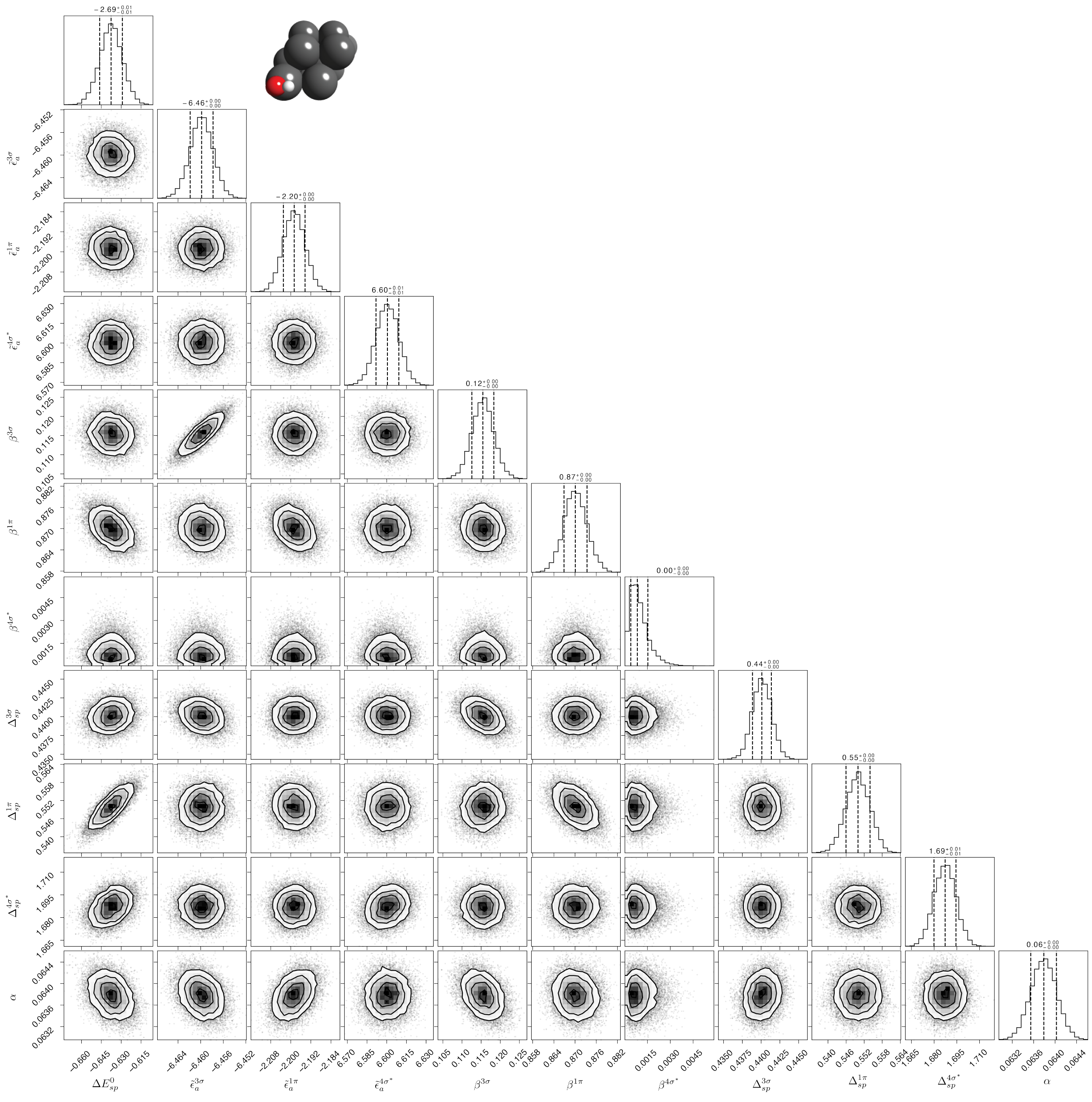

Supplementary Figure 8. The co-variance of the joint posterior distribution for each parameter pair and 1D histogram of model parameters (ΔE_0, $\varepsilon_a^{3\sigma}$, $\varepsilon_a^{1\pi}$, $\varepsilon_a^{4\sigma^*}$, $\beta^{3\sigma}$, $\beta^{1\pi}$, $\beta^{4\sigma^*}$, $\Delta_0^{3\sigma}$, $\Delta_0^{1\pi}$, $\Delta_0^{4\sigma^*}$, and α) from MCMC simulations for *OH adsorption at the atop site of the close-packed, pristine transition-metal surfaces.

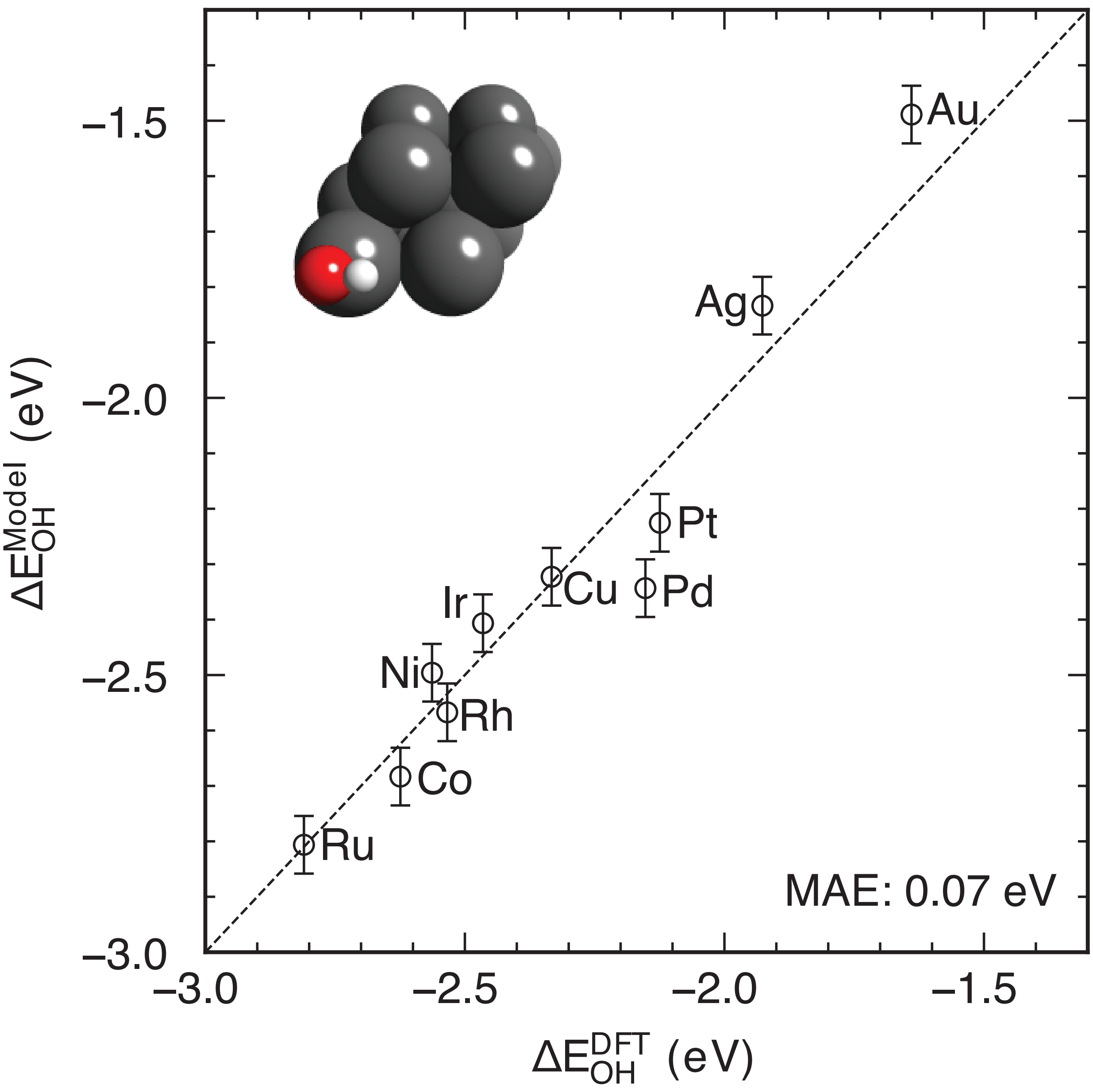

Supplementary Figure 9. DFT-calculated *OH adsorption energies at metal surfaces vs. model prediction using the posterior distribution of model parameters ($\vec{\theta}$, $\vec{\sigma}$). Error bars represent the standard deviation of prediction based on Bayesian sampling.

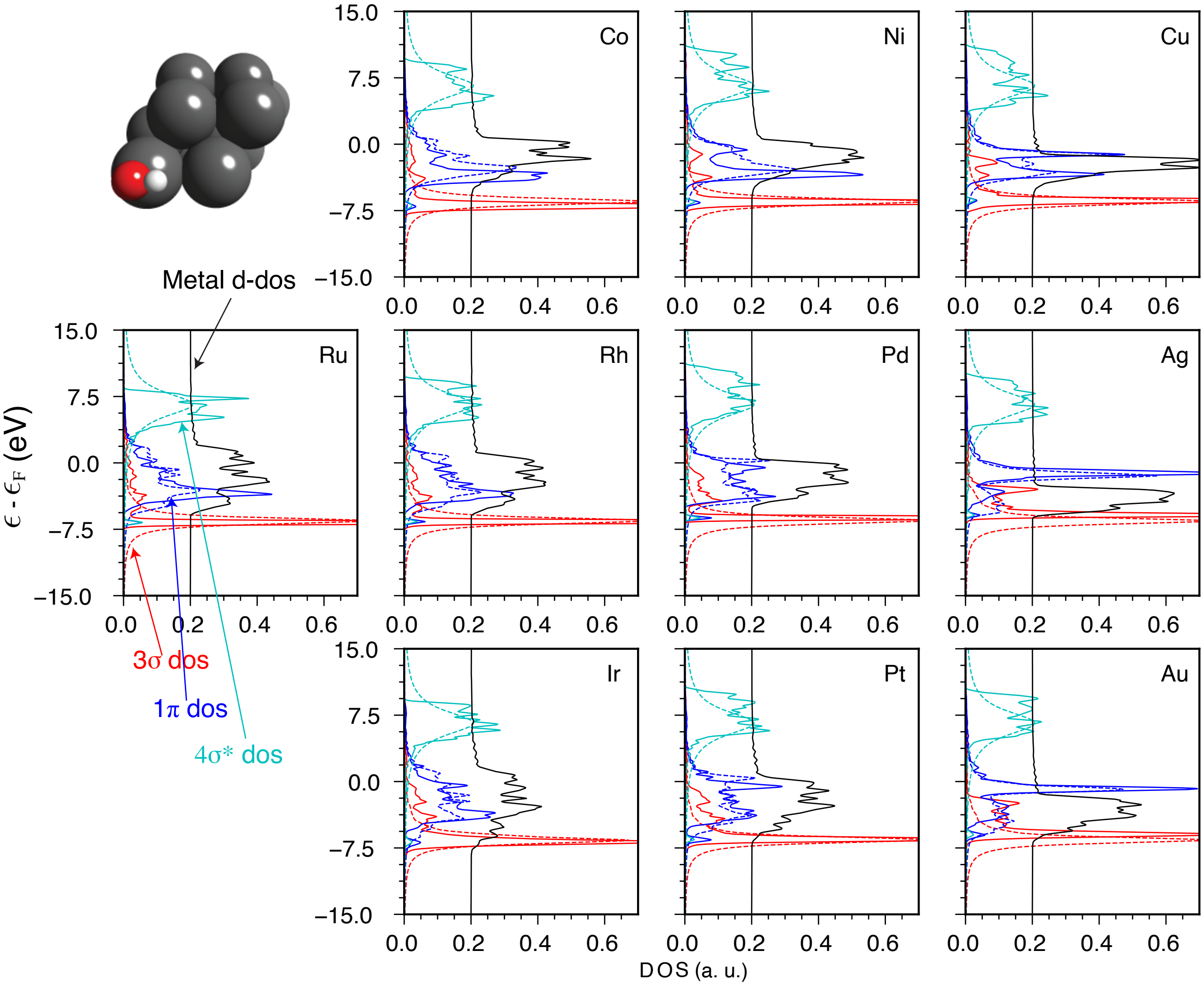

Supplementary Figure 10. Symmetry resolved projected molecular orbital density of states onto *OH from DFT calculations (solid) and model prediction (dashed) using the Bayesian-learned posterior means of model parameters. Metal *d*-states are also shown.

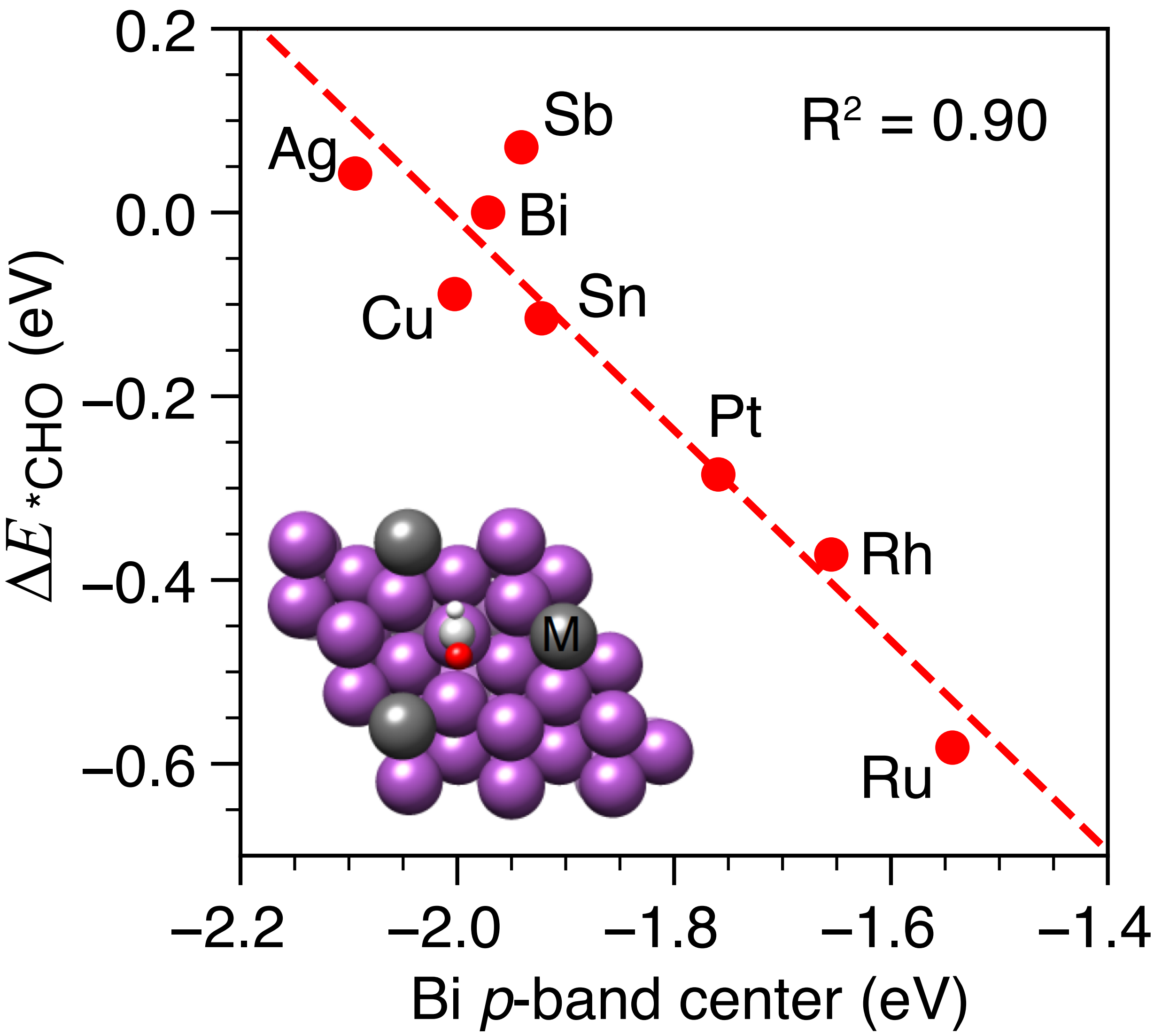

Supplementary Figure 11. The adsorption energy of *CHO at the atop Bi site of M-doped Bi(001) surfaces vs. the Bi *p*-band center of all states. The inset shows model structures of M-doped Bi(001).

Supplementary References

[1] P. Giannozzi, S. Baroni, N. Bonini, M. Calandra, R. Car, C. Cavazzoni, D. Ceresoli, G. L. Chiarotti, M. Cococcioni, I. Dabo, A. Dal Corso, S. de Gironcoli, S. Fabris, G. Fratesi, R. Gebauer, U. Gerstmann, C. Gougoussis, A. Kokalj, M. Lazzeri, L. Martin-Samos, N. Marzari, F. Mauri, R. Mazzarello, S. Paolini, A. Pasquarello, L. Paulatto, C. Sbraccia, S. Scandolo, G. Sclauzero, A. P. Seitsonen, A. Smogunov, P. Umari, and R. M. Wentzcovitch, J. Phys. Condens. Matter **21**, 395502 (2009).

[2] J. P. Perdew, K. Burke, and M. Ernzerhof, Phys. Rev. Lett. **77**, 3865 (1996).

[3] Z. Li, S. Wang, W. S. Chin, L. E. Achenie, and H. Xin, J. Mater. Chem. A Mater. Energy Sustain. **5**, 24131 (2017).

[4] A. Patil, D. Huard, and C. J. Fonnesbeck, J. Stat. Softw. **35**, 1 (2010).

[5] D. Gamerman and H. F. Lopes, *Markov Chain Monte Carlo: Stochastic Simulation for Bayesian Inference, Second Edition (Chapman & Hall/CRC Texts in Statistical Science)*, 2nd ed. (Chapman and Hall/CRC, 2006).
